# The Power of Electropenetrography in Enhancing Our Understanding of Host Plant-Vector Interactions

**DOI:** 10.3390/insects10110407

**Published:** 2019-11-15

**Authors:** Holly Shugart, Timothy Ebert, Frederick Gmitter, Michael Rogers

**Affiliations:** 1 Department of Plant Pathology, Citrus Research and Education Center, University of Florida, 700 Experiment Station Road, Lake Alfred, FL 33850, USA; 2 Department of Entomology and Nematology, Citrus Research and Education Center, University of Florida, 700 Experiment Station Road, Lake Alfred, FL 33850, USA; tebert@ufl.edu (T.E.); mrgrs@ufl.edu (M.R.); 3 Department of Horticulture Science, Citrus Research and Education Center, University of Florida, 700 Experiment Station Road, Lake Alfred, FL 33850, USA; fgmitter@ufl.edu

**Keywords:** EPG, electrical penetration graph, GC-MS, gas-chromatography mass-spectrometry, bacteria-insect interactions

## Abstract

The invasive Asian citrus psyllid, *Diaphorina citri* (Hemiptera: Liviidae), is the primary vector of the phloem-infecting bacterium, *Candidatus* Liberibacter asiaticus. *Candidatus* L. asiaticus is the putative causal agent of Huanglongbing (HLB) disease, a destructive disease of *Citrus*. While many *Citrus* species are susceptible to *D. citri* probing and HLB disease, there are marked behavioral differences in *D. citri* probing responses and Ca. Liberibacter asiaticus infection severity among *Citrus* species. Using four mandarin hybrid selections and pummelo plants variably resistant to *D. citri* probing, oviposition, and survival, we explored probing differences using electropenetrography (EPG), conducted an oviposition and survival study, and determined host plant metabolites using gas-chromatography mass-spectroscopy (GC-MS). We found thirty-seven *D. citri* probing variables to be significantly different among tested mandarin selections and pummelo, in addition to differential oviposition and survivorship abilities on tested plants. We found sixty-three leaf metabolites with eight being significantly different among tested mandarin selections and pummelo. Detailed analysis of probing behavior, oviposition, survivorship, and host plant metabolite concentrations reveals the complex, layered resistance mechanisms utilized by resistant *Citrus* against *D. citri* probing. EPG is a powerful technology for screening Asian citrus psyllid resistant *Citrus* to elucidate host plant-vector interactions, with an aim to minimize vector probing and eliminate the spread of the bacterial pathogen, Ca. L. asiaticus.

## 1. Introduction

The Asian citrus psyllid, *Diaphorina citri* Kuwayama (Hemiptera: Liviidae), is an invasive insect in North America and primary vector of the phloem-infecting gram-negative α-proteobacterium, *Candidatus* Liberibacter asiaticus [1]. *Diaphorina citri* feeds on and transmits Ca. L. asiaticus to many *Citrus* species and to several other non-agricultural or ornamental plants within the plant family Rutaceae [2]. Huanglongbing (HLB), caused by Ca. L. asiaticus, is a devastating and incurable disease of citrus. HLB has quickly become the most economically important disease of production citrus in North America since the arrival of the pathogen in 2005 [3,4].

Many *Citrus* species and varieties are susceptible to *D. citri* probing and to HLB disease. *Diaphorina citri* probing behaviors differ among *Citrus* species and other hosts within the Rutaceae (*C. sinensis*-[5], *C. reticulata*-[6], *C. sunki*-[7]). While it is critical to understand the probing behavior of hemipteran pests and vectors, these behaviors cannot be easily observed and quantified, since the insects insert their piercing-sucking mouthparts into opaque plant tissues such that the subsequent probing activities are invisible to observers. The most precise method of quantifying the probing behavior of hemipteran pests and vectors is electropenetrography (EPG). EPG requires the attachment of conductive gold wire to the pronotum of the insect and the application of a small electrical signal to the host plant to complete an electrical circuit and generate a waveform (voltage by time) [8]. Probing waveforms are produced any time the insect inserts the stylets into plant tissues. Waveforms are consistent and reproducible such that salivation, stylet movements, the puncturing of plant cells, and ingestion behaviors can all be observed and quantified [9,10].

Despite years of investigation, the specific mechanisms of *Citrus* host plant resistance to *D. ctiri* probing eludes researchers, and no comprehensive method has been employed to screen host plants for resistance to vector probing and pathogen transmission. Many investigations into the probing behavior of a pest on variable hosts can discover probing differences. However, placing those behavioral differences into context is crucial to understanding the underlying mechanisms of host plant resistance. What are the specific characteristics of each host influencing vector probing behavior? Does the host employ physical or chemical feeding deterrents, are there physical impediments to probing or accessing the phloem, does the phloem contain chemical defenses, and can the vector insect successfully oviposit and develop on the host? To gain a comprehensive view of the total vector-host plant interactions, researchers must combine data obtained from other powerful techniques together with the observed probing differences. The most comprehensive EPG studies combine an investigation of probing behavior with other powerful techniques, such as GC-MS [11,12,13,14], RNAi techniques [15], micro-CT [16], and use a combination of robust univariate and multivariate statistics [5,17,18,19,20] to link causal factors to the observed probing differences and obtain a more complete picture of the probing interactions of *D. citri* with each tested host.

In order to assign resistance levels to *D. citri* probing in previously untested potentially resistant mandarin selections with pummelo as a susceptible outgroup, we combined a robust investigation of probing behavior, a survey of oviposition and survival, and the determination of leaf metabolites for each host. We expected that the life history data and leaf metabolites would mirror the EPG probing profiles and explain many aspects of *D. citri* probing behaviors on variably resistant *Citrus* hosts. In order to achieve a baseline probing profile for each host, we chose to record pathogen-free psyllids. Despite this, much of the analysis was completed with the transmission mechanism of the phloem-infecting Ca. Liberibacter pathogen in mind. As such, we focused our analysis on how successfully *D. citri* interacted with the phloem. We calculated variables focused on determining the exact moment the psyllid reaches the phloem, how many different times the phloem was accessed, durations spent in phloem tissues, and how much of the time spent in phloem included ingestion behaviors. This study demonstrates the capacity of the EPG technique to elucidate the details of probing differences of even closely related host plants. The mandarin selections tested here were closely related, all sharing a single pollen donor parent, and yet the range of probing responses discovered was striking. EPG is a powerful screening tool for determining probing responses of vectors of plant pathogens to resistant species and selections.

## 2. Materials and Methods

### 2.1. Plants

Crosses were made at the Citrus Research and Education Center in Lake Alfred, Florida, USA, using variable *Citrus reshni* as the female parents. *Citrus ichangensis* pollen was obtained from a single plant at the Florida Citrus Arboretum in Winter Haven, FL, USA. Resulting seedlings were grafted onto *Carrizo citrange* rootstock. Pummelo plants were not grafted.

Plants were 5–6 years old during this experiment and were maintained in a greenhouse with natural lighting with fans turning on at 30 °C. There was no additional heating, cooling, or supplemental lighting. Plants were fertilized with either Harrell’s Pro-fertilizer 12-3-8, Harrell’s LLC, Lakeland, FL, USA) or Miracle Gro All Purpose plant food (Scotts Miracle Gro^®^, Marysville, OH, USA). Plants were pruned regularly to promote the production of new growth.

### 2.2. Insects

Test psyllids were removed from a Ca. Liberibacter-free Curry Leaf-based colony started in 2006 and maintained consistently in a growth room at the Citrus Research and Education Center in Lake Alfred, FL, USA. Psyllids were maintained on Curry Leaf, *Bergera koengii* (formerly *Murraya koengii*), and prior to EPG recording, psyllids were acclimated to the host plant on which they would be tested for at least one week within Bugdorm^®^ cages (Bioquip Products, Rancho Dominguez, CA, USA). Mandarin and pummelo psyllid colonies were maintained under natural lighting conditions within an outdoor screen cage just a few feet from the greenhouse in which the test plants were maintained. Colony plants and insects were exposed to natural rainwater and given supplemental water as needed. The experiment began one week after the mandarin and pummelo colonies were established and continued for three months. Several of the mandarin colonies had to be re-stocked with *Bergera* colony psyllids because psyllids could not reproduce well on the plants and the experiment was halted to allow for a one-week acclimation period.

### 2.3. Electropenetrography Equipment and Settings

**Figure 1:**
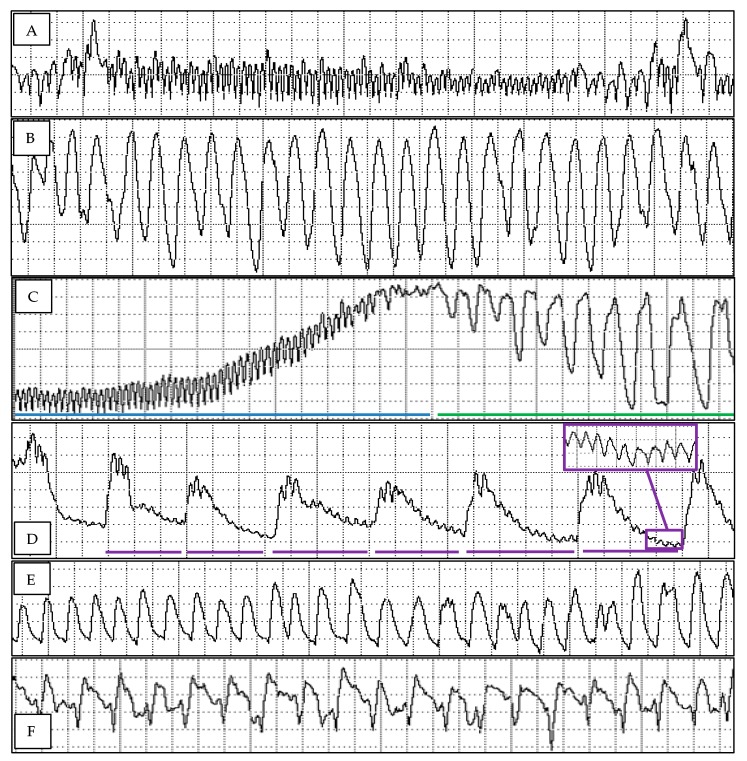
Waveform excerpts of *Diaphorina citri* probing four Cleopatra Mandarin selections and Pummelo. (**A**) Waveform C representing probing epidermis, parenchyma, and cambium tissues *en route* to vascular tissues. 0.27 s/div. (**B**) Waveform G representing ingestion from xylem tissues. 0.13 s/div, peak frequency 0.15 s. (**C**) The first portion of waveform D showing the voltage rise (blue line) from waveform C and the tall, narrow peaks (green line) that immediately follow waveform C. 0.27 s/div. (**D**) The later portion of waveform D showing the lower voltage, and lower frequency peaks (each with a purple line underneath, peak frequency 2.67 s) with smaller, higher frequency peaks superimposed. These smaller, high frequency peaks (purple inset box) will grow into higher voltage peaks that will become the first peaks of waveform E1. 0.27 s/div. (**E**) Waveform E1 representing salivation in phloem tissues. 0.13 s/div, peak frequency 0.133 s. (**F**) Waveform E2 representing ingestion from phloem tissues. 0.13 s/div, peak frequency 0.19 s.

Psyllids were wired by affixing a 0.001” (25.4 µm) diameter gold wire (Sigmund Cohn Corp., Mount Vernon, NY, USA) to the pronotum of the insect using water-based silver glue (1 mL water, 1 mL water-based glue, 1 g silver flake). Psyllids were allowed to dangle from their wires for 30 min–1 h prior to the recording period, which lasted twenty-four hours. The final sample size for each treatment was as follows: Pummelo (21 psyllids), mandarin 2 (22 psyllids), mandarin 19 (23 psyllids), mandarin 26 (20 psyllids), and mandarin 31 (21 psyllids). Psyllids were given access to the abaxial leaf surface during the recording and were initially positioned near the midvein, as this is where they prefer to probe [5,21]. Psyllids were recorded on immature leaves with the leaf sizes ranging from leaf #5–12 from Figure 1 in Reference [5].

Recordings were made using two four-channel analog AC-DC EPG monitors built by EPG Technologies, Inc. Gainesville, FL, USA per the design outlined in References [8,22]. Recordings were made using an input impedance of 10^9^ Ohms with an applied DC voltage of 150 mV, a voltage shown to have no detectable effect on psyllid probing [17]. Signals were digitized using a DI710 converter (Dataq Instruments, Akron, OH, USA) and waveforms were recorded and measured using Windaq software (Dataq Instruments, Windaq Lite for acquisition and Windaq Waveform Browser for post-acquisition visualization and measurement). Psyllid waveform names were established and partially correlated by Bonani, et al. [23], and the analysis herein follows the naming convention, assigned behaviors, and stylet tip locations established in Bonani, et al. [23], and further investigated in Shugart [24]. Waveforms measured included pathway (C), phloem contact (D), phloem salivation (E1), phloem ingestion (E2), xylem ingestion (G), and a non-probing waveform (NP). The lighting environment of the room included overhead fluorescent fixtures kept on during recording (24:0 light:dark ratio) with no natural light input. The temperature was maintained between 25–28 °C.

### 2.4. Electropenetrography Data Analysis

Waveform data were analyzed using the Ebert 2.0 SAS program [25], which is freely available on the web at (https://crec.ifas.ufl.edu/extension/epg/). SAS^®^ version 9.4TS1M was used within the SAS^®^ enterprise guide version 7.15HF3 for this analysis. The Ebert program calculates 89 variables (a complete list can be found in the supplemental information of Ebert, et al. [25]) and then run an ANOVA in proc GLIMMIX model ANOVA. Means were separated using an LSD test, with the *p*-value set to 0.05. All EPG variable data were presented as untransformed.

### 2.5. Ranking Resistance Levels in Mandarin Selections and Pummelo

The means of twenty-four of the thirty-seven Ebert 2.0 program variables found to be significantly different among treatments were ranked along a continuous scale from one to five. The higher value was given to the mean indicating the most resistant value associated with each variable. The twenty-four variables used were chosen because means, representing psyllid behaviors, could be associated with a behavioral trend indicating either resistance or susceptibility of the host to *D. citri* probing behavior based on previous EPG studies [5,17,26]. For example, the highest value for variable TtlDurNP, or the total duration of the recording spent not probing, was given a rank of five, while the lowest value was given a rank of one. The highest value for this variable indicates that a large portion of the recording period was spent not probing and was associated with resistance to *D. citri* probing. In some cases, the largest value was interpreted as an indicator of susceptibility. For example, NumLngE2, or the number of long waveform E2 events (>10 min), means were ranked such that the largest value was given a rank of one, while the smallest value was given a rank of five. The significance of the means of each variable was carefully considered in this way so the means of twenty-four variables could be ranked along a continuum of resistance to susceptibility, then the ranks from each variable summed to get an overall resistance rank for each tested selection or species.

### 2.6. Discriminant Analysis

The univariate analysis provides a detailed assessment of the differences in vector responses to variably resistant hosts. However, it is helpful to visualize a broader picture of the overall differences in vector responses to these hosts. To this end, we used a stepwise discriminant analysis (procStepDisc) as a variable reduction technique. The selected variables (CtoFrstG, TmFrstSusE2FrstPrb, DurNnprbBfrFrstG, NumE2, PrcntPrbD, PrcntPrbC, TtlDurC, and PrcntPrbG) were then used in a canonical discriminant analysis (proc CanDisc), and part of that analysis calculated a unitless measure of the distance between treatments called a Mahalanobis distance. The upper triangular matrix is the Mehalanobis distances between *D. citri* behavior on two compared hosts, while the lower triangular matrix is a *p*-value testing the null hypothesis that the distance between the compared behaviors is zero. Recordings were not included in the model if a behavior necessary to calculate each variable was not performed by the psyllid, so that 104 of the 107 psyllids recorded were used in the calculated model.

### 2.7. Transitional Probabilities and Kinetograms

Transitional probabilities were calculated using the Ebert 2.0 SAS program and are summarized in behavioral kinetograms. The arrows between squares represent the frequency of transitional events for each waveform per treatment, and the direction of the arrow indicates the preceding waveform (beginning of the arrow) and the waveform that follows (pointer of the arrow). Several arrows may originate from a single box (waveform type) if the waveform type can transition to multiple waveform types. All of the arrows that originate from a single box add up to 100%. These data are sums of all transitions occurring per treatment, and therefore, are not appropriate for statistical analysis.

### 2.8. Leaf Metabolites

Three leaves (one young, one medium-aged, and one mature) were excised from 5 plants of each of the mandarin selections and pummelo. Leaves were sampled three days after the completion of the EPG portion of the experiment with the aim of acquiring a representative snapshot of what the metabolites were during the course of the experiment, but not link the specific metabolite profiles of individual plants with the probing behaviors of EPG-recorded psyllids. Metabolites were analyzed from 0.1 g (fresh leaf weight), from three pooled leaf samples from five biological replicates of each treatment for metabolite analysis (*n* = 5). Leaf tissue was homogenized in liquid Nitrogen, then extracted with 1 mL extraction solvent (8:1:1 methylanol, chloroform, water). Ribitol was used as an internal standard with a concentration of 100 ppm in the final derivatized sample. Dried residues were derivatized with a trimethylsilylation (TMS) procedure using 30 mL of methoxyamine hydrochloride solution (MOX) in pyridine (2%) and allowed to react for 17 h at room temperature. At the end of the methoximation, the sample was mixed with 80 mL of N-methyl-(N-trimethylsilyl) trifluoracetamide (MSTFA) and left for 2 h at room temperature and processed using GC-MS conditions as described in Killiny et al. [27]. Injection volume was 0.5 µL. All peak areas were normalized to the mean area of the internal standard and converted to µg/g using calibration curves of authentic standards for each compound type were derivatized and injected into the GC-MS in the same way as experimental samples. Compounds not detected were noted as ND and zeros (0.0) were removed from the means calculations.

### 2.9. Metabolite Statistical Analysis

Plant metabolite concentrations were determined for sixty-six compounds. Mean concentrations were analyzed with SAS analysis software, using ANOVA and means were separated using an LSD test set to *p* = 0.05. Values below the detection limit of the GC-MS method were treated as zeros. All data are presented as untransformed. The degrees of freedom for all leaf metabolite data is 4, because five plants (biological replicates) were sampled to generate these data.

### 2.10. Diaphorina citri Oviposition and Survivorship on Mandarin Selections and Pummelo

To determine if oviposition and survivorship mirrored probing ability on each selection or species, a no choice oviposition study was performed, and the survivorship of eggs to adulthood was observed on the four mandarin selections and pummelo. The psyllids used for this experiment were Ca. Liberibacter-free and were from the same sources as those used in the EPG experiment. Plants were maintained in a greenhouse environment under the same environment as the plants used for the EPG study described previously. Plants were chosen for use in this experiment based on the availability of young leaf growth, as this is tissue on which *D. citri* oviposits [28]. Three plants were selected with a range of sizes of young flush, placed in a Bugdorm^®^ cage (Bioquip Products, Rancho Dominguez, CA, USA) with 25 females and 25 males, allowing psyllids to move freely and choose the size of young leaves preferred for feeding and oviposition. Each cohort of psyllids was provided with plants bearing at least ten appropriately sized groups of young leaves (mandarin 2-*n* = 16, mandarin 19-*n* = 10, mandarin 26-*n* = 10, mandarin 31-*n* = 12, and pummelo-*n* = 16). Tests were performed in a growth room with artificial lighting (14:10 light:dark ratio), with the temperature maintained between 25–30 °C, and humidity kept between 60–80%.

Psyllids were allowed to mate and oviposit freely for 10 days, at which point the adult psyllids were removed. During development, young psyllid nymphs (first through third instars) are nearly sessile, allowing for quantification of nymphs from each group of young leaves as nymphs were not moving from one group to another. During the fourth and fifth instars, *D. citri* can be mobile. However, they were not observed moving between different groups of young leaves. Groups of young leaves were separated by long sections of the woody stem in most cases. The immobility of the nymphs in the current study allowed for the quantification of the development of nymphs on individual sets of young leaves. As adults emerged, they were removed daily to prevent mating and further oviposition. The number of eggs laid per treatment during the 10 day oviposition window was summed for each treatment and survivorship counted at several time points during development (days 20, 24, 27, and 29) until all individuals had become adults (and were removed) or had died since the previous count. The percentage of eggs laid that survived to adult was calculated. Confidence intervals were calculated using the R (https://www.r-project.org/version3.5.3) binGroup package (https://www.rdocumentation.org/packages/binGroup) running in RStudio (https://www.rstudio.com/version1.1.456).

## 3. Results

### 3.1. Diaphorina citri Probing Profiles on Mandarin Selections and Pummelo

Thirty-seven of the eighty-nine variables calculated showed a significant treatment effect (Table 1), and representative waveforms from this study can be found in Figure 1. These variables largely focused on the sequence and timing of when psyllids reach the phloem and xylem and how long they spent performing phloem and xylem behaviors. The timing and sequence of waveforms performed, and the location of the stylets during the performance of each waveform can provide insights as to the type of resistance factors affecting the probing behaviors on a tested host. We found resistance factors impacting *D. citri* probing behavior at all levels of probing, within the cuticle and epidermis, parenchyma tissues *en route* to the phloem, at the edge of the phloem, and within the phloem sap. The most resistant mandarin selections, 31 and 2, had resistance factors operating at all of these levels of probing, while *D. citri* probed more successfully on mandarin 19 and pummelo. Behavioral profiles were developed to describe the cohort of probing behaviors exhibited by *D. citri* on each host.

The behavioral profile of *D. citri* on mandarin 31 indicated resistance factors at every level during the probing process: During the initiation of probing, while stylets pass through parenchyma tissues, phloem access, and maintenance of phloem ingestion. Psyllids took longer to make the initial probe (TmFrstPrbFrmStrt-0.1 h) during the recording period and spent the most time not probing (TtlDurNP-7.9 h) on mandarin 31. These variables indicate resistance factors affecting the beginning of the probe, either in the cuticle or epidermis, that prevent the psyllid from initiating a probe. Psyllids probing mandarin 31 also exhibited a reduction of non-probing occurring before the first performance of waveform E1 (DurNnprbBfrFrstE1-3.8 h) among tested hosts.

Psyllids probing mandarin 31 also struggled during their initial contact with phloem tissues. During waveform D, psyllids taste and salivate into a phloem sieve element, and make important decisions about the acceptability of the cell. Longer waveform D events and longer periods overall spent performing waveform D represent the difficulty in finding an acceptable phloem sieve element from which to begin phloem ingestion. The behavioral profile of psyllids probing mandarin 31 includes the highest values for four of the six statistically significant waveform D associated variables, including: NumLngD, maxD, meanD, and PrcntPrbD. The number of long D waveform events lasting 100 s or more occurred 2.4 times per insect on mandarin 31, but only 0.2 and 0.3 times per insect on pummelo and mandarin 26, respectively. Psyllids probing mandarin 31 also performed the longest duration of a single D waveform event (maxD-142 s), the longest mean duration of waveform D (meanD-90 s), and spent the largest percent of total probing time performing waveform D (PrcntPrbD-10.3%). Psyllids also struggled to maintain phloem ingestion, while probing mandarin 31. The longest phloem ingestion event (maxE2) on mandarin 31 was 2.3 h long, the shortest of all on tested host plants. Psyllids spent the shortest time overall performing phloem ingestion (TtlDurE2-4.7 h) on Mandarin 31. *Diaphorina citri* struggled both to access phloem tissues and maintain phloem ingestion, while probing mandarin 31.

The behavioral profile of *D. citri* probing mandarin 2 indicates resistance factors within the parenchyma, and factors influencing both phloem access and the psyllid’s ability to maintain phloem ingestion. Unlike when psyllids probed mandarin 31 the initiation of probing was delayed, psyllids probed mandarin 2 more quickly. In fact, psyllids probing mandarin 2 had the shortest non-probing duration (TtlDurNP-4.7 h) of all tested hosts. However, once probing commenced on mandarin 2, psyllids spent long durations passing through the parenchyma to reach the phloem. While probing mandarin 2, psyllids performed the longest duration of waveform C (TtlDurC-11 h), representing time spent with stylets in parenchyma tissues, and the longest duration before the first waveform D was performed during the recording (TtlNnprbBfrFrstD-2.9 h). Together, these variables represent an increased duration of time spent probing parenchyma tissues, resulting in a delayed and reduced duration of time spent probing phloem tissues.

The behavioral profile of *D. citri* probing mandarin 2 indicates substantial difficulty in accessing the phloem tissues. The time that passes before phloem access is made up of the time spent not probing, the time spent probing parenchyma tissues (waveform C) and the time spent and the number of waveform events of waveform D, and at times the performance of xylem ingestion (waveform G), which psyllids will often do when they cannot access or maintain phloem ingestion. In the case of psyllids probing mandarin 2, several variables are associated with difficulty accessing the phloem, including: An increased time to the first sustained E2 waveform (TmFrstSusE2-15.1 h), an increased time from the first probe to the first E waveform event (TmFrmFrstPrbFrstE-12.3 h), and increased time to the first E2 waveform from the start of the recording (TmFrstE2StrtEPG-13.8 h), and an increased time to the first sustained E2 waveform from the start of the first probe (TmFrstSusE2FrstPrb-15 h). Psyllids probing mandarin 2 struggled to maintain phloem ingestion and especially struggled to maintain sustained phloem ingestion, defined as phloem ingestion events lasting longer than 600 s. While probing mandarin 2, psyllids spent the smallest percentage of phloem ingestion performing sustained phloem ingestion (PrcntE2SusE2-44%) and performed the smallest number of long E2 waveform events (NumLngE2-1.3) compared to other tested hosts. Additionally, psyllids spent the largest duration ingesting from xylem (DurG-3 h), while probing mandarin 2, a behavioral switch performed when psyllids cannot successfully access or maintain phloem ingestion.

The behavioral profile of *D. citri* probing mandarin 26 indicated resistance factors to probing is minimal from the cuticle, epidermis, and edge of the phloem, but are clearer for behaviors performed in parenchyma tissues and within the phloem sieve elements. Evidence of resistance factors in the parenchyma include an increased time to the first performance of waveform D within each probe (TmFrmFrstPrbFrstD-7.8 h), an increased duration of the total time not spent with stylets in phloem tissues (TtlDurNnPhlPhs-19.1 h), as well as the fact that psyllids spent 62% of probing time performing waveform C primarily within parenchyma tissues. Once the phloem tissues were accessed, psyllids struggled to maintain phloem ingestion, with the smallest percentage of phloem ingestion of total probing duration (PrcntPrbE2-19.5%) occurring as psyllids probed mandarin 26.

The behavioral profile of *D. citri* probing mandarin 19 was not strongly linked to any resistance-associated behaviors. Rather, psyllids initiated probing quickly, spent an average time performing waveform C in parenchyma tissues, accessed the phloem relatively easily, spent the most time in phloem (TtlDurE2-10.4 h), and performed the largest percentage of time ingesting from phloem of the total probing duration (PrcntPrbE2-57%). In fact, psyllids probing mandarin 19 spent nearly three times longer ingesting from phloem compared to psyllids probing the most resistant selection, mandarin 31, with only 22.5% probing time spent in phloem.

The behavioral profile of *D. citri* probing pummelo was not strongly linked to any resistance-associated behaviors. Rather, psyllids readily probed, reached the phloem quickly, and spent long durations performing sustained phloem ingestion. Psyllids probing pummelo spent the least amount of time passing through parenchyma tissues (TtlDurC-7.9 h), and once the phloem was contacted, psyllids performed the shortest waveform D (meanD-43 s), allowing them to begin phloem salivation and ingestion quickly (TmFrstE2StrtEPG-7.4 h). Once phloem ingestion began, psyllids spent relatively more time ingesting phloem (PrcntPrbE2-37%) and the most time performing sustained phloem ingestion (PrcntE2SusE2-72%) of time spent ingesting phloem sap overall.

### 3.2. Ranking Resistance Levels in Mandarin Selections and Pummelo

The continuous ranks assigned to the means of twenty-four variables from each host plant treatment were summed and a total value determined as a way to quantify the level of resistance. The largest total rank value was equated with a higher resistance level. The host plant with the largest total rank value was mandarin 31 with a value of 94, making it the most resistant treatment. Mandarin 2 was ranked second most resistant with a total rank of 87. Mandarin 26 was ranked third most resistant with a total rank of 84. Mandarin 19 was the least resistant of the mandarin selections with a total rank value of 54. Pummelo was ranked as the most susceptible host plant treatment in this study with a total rank value of 40. The complete list of variables used, and associated ranks of means are summarized in Table 1 and Table 2.

### 3.3. Transitional Probabilities and Kinetograms

**Figure 2:**
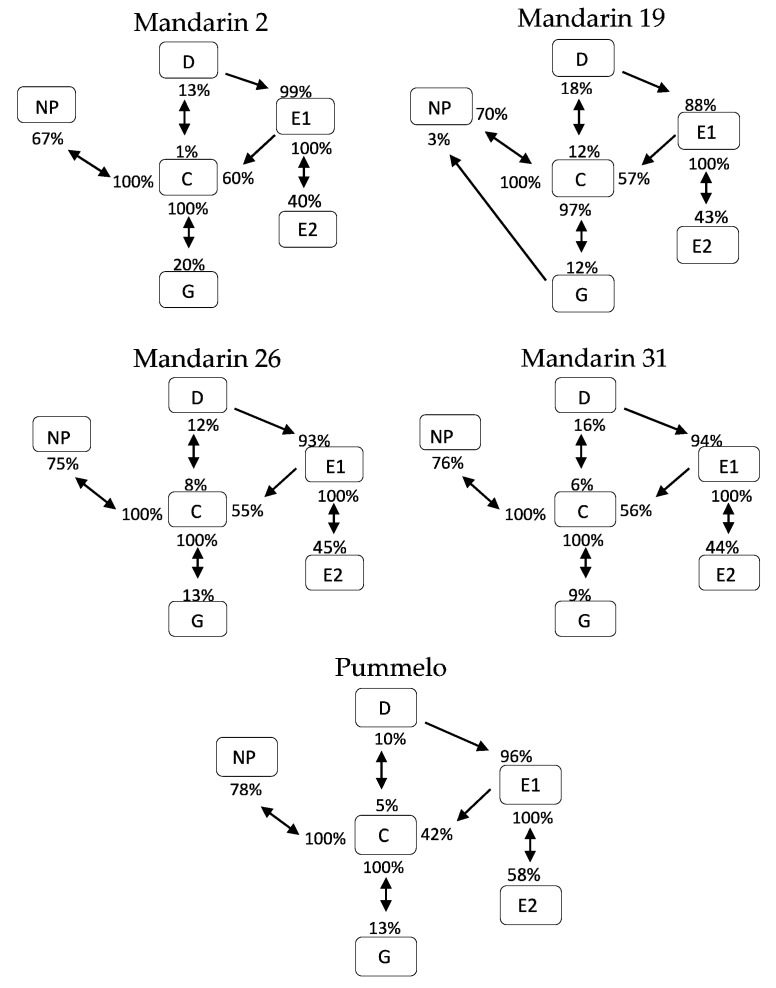
Kinetogram of behavioral transitions of *Diaphorina citri* probing four Cleopatra Mandarin selections and Pummelo.

Transitional probabilities are the frequency of occurrence of a waveform type following in sequence from the previously performed waveform type. The transitional probabilities for host plants recorded as part of this study are summarized in kinetograms for each host (Figure 2). Not all waveform types can follow each waveform type. For example, the non-probing waveform (NP) can only be followed by waveform C. Waveform C can transition into waveform D, G, or back to NP. Waveform D can transition to waveform E1, or C. Waveform E1 can transition to waveform E2 or to C. Waveform E2 always transitions to E1. Waveform G almost always transitions to waveform C, but can rarely transition directly to waveform NP, as occurred 3% of transitions, while probing mandarin 19 in this study.

While these summed data are not appropriate for statistical comparisons between treatments, patterns in transition frequencies can be observed and represent the range of ways that *D. citri* interacts with different hosts. For example, pummelo differed from other hosts in that waveform E1 more frequently transitioned to waveform E2 (58%) compared to the transition rate of 40–48% in the other tested hosts. Pummelo also differed from other hosts in the rate that waveform E1 transitioned to waveform C (42%), less frequently than on mandarin hosts where the rate was 55–60%. The transition rates from waveform E1 to either waveform C or waveform E2 are correlated, as waveform C and waveform E2 are the only possible options. Another observed trend was that the transitions from waveform C to waveform G in mandarin 2 were higher than observed in other hosts at 20%, with this transition occurring in other hosts at a frequency ranging from 9–13%. Mandarin 19 differed from other hosts in the rate at which waveform D transitioned to waveform E1, with waveform D being followed by waveform E1 88% of the time compared to at a rate ranging from 93–99% in other hosts.

Waveform types are arranged within squares with percentages near the arrows representing the probabilities of each waveform transitioning to another waveform.

### 3.4. Discriminant Analysis

Each cell of the table represents *D. citri* probing responses summarized by eight variables (outlined in the methods) to two compared hosts (Table 3). Larger values in the upper triangle of the matrix represent a larger distance between the two compared hosts, meaning psyllid responses are not similar for each compared host. The outcomes of the discriminant analysis closely match the ranking comparisons (Table 2). Pummelo is most different from the mandarin hosts, especially mandarin 19 (5.97) and mandarin 31 (5.55). Mandarin 31 (most resistant) and mandarin 19 (most susceptible of the mandarins) had high distances (5.59) when compared to one another. The lower triangular matrix is a p-value testing the null hypothesis that the distance between the compared behaviors is zero (Table 3). All comparisons of two different hosts were statistically significant at *p* = 0.05 with one exception, mandarin 2 and mandarin 26, at 0.2727.

### 3.5. Metabolite Profiles

**Figure 3:**
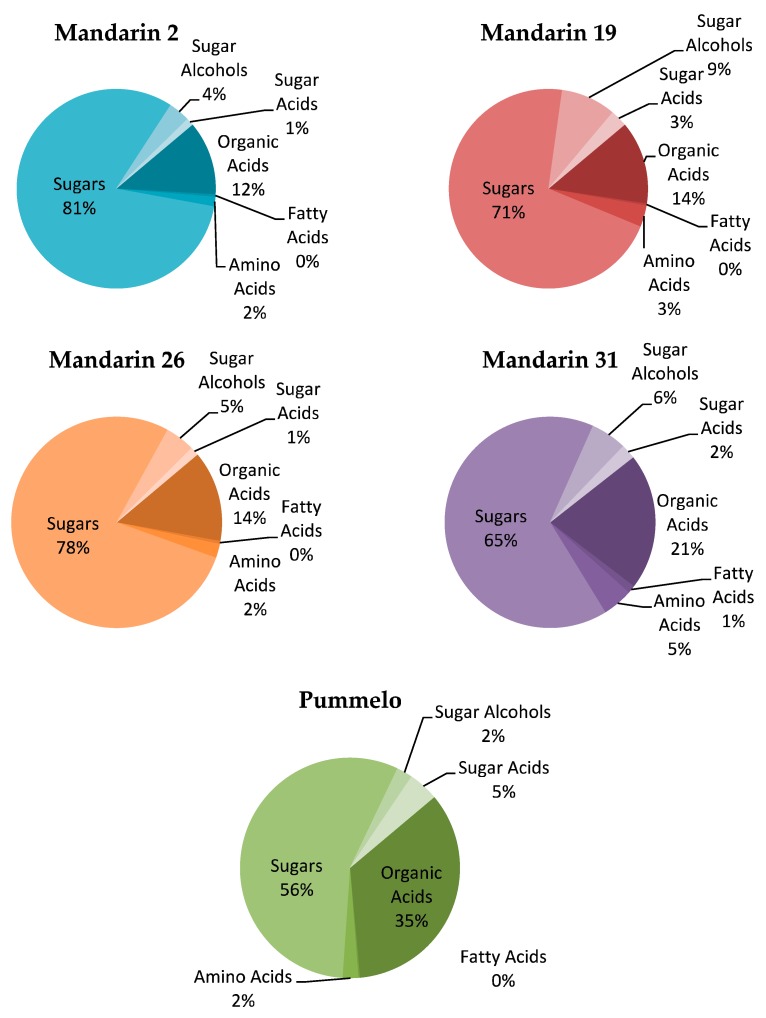
Percentage composition of metabolic groups found in four Cleopatra Mandarin selections and Pummelo.

A total of sixty-three metabolites were found in the mandarin selections and pummelo. A total of eight metabolites were found in statistically different concentrations among treatments (Table 4, Figure 3). The amino acid, serine, was found in the highest concentration in pummelo, 187.76 µg/g, and in the lowest concentration in mandarin 19, 18.02 µg/g (*p* = 0.0173). Two organic acids, succinic acid and quinic acid, were found in statistically different concentrations. Succinic acid was found in the highest concentration in pummelo, 12.99 µg/g, and in the lowest concentration in mandarin 19, 1.18 µg/g (*p* = 0.0297). Quinic acid was found in the highest concentration in mandarin 2, 9.75 µg/g, and in the lowest concentration in mandarin 26, 4.17 µg/g (*p* = 0.0203). The fatty acid, oleic acid, was found in the highest concentration in mandarin 19, 2.44 µg/g, and in the lowest concentration in mandarin 31, 0.4 µg/g (*p* = 0.0072). Two sugars, xylose 1 and α-galactose, were found in significantly different concentrations. Xylose 1 was found in the highest concentration in pummelo, 16.78 µg/g, and in the lowest concentration in mandarin 31, 3.08 µg/g (*p* = 0.0473). α-galactose was found in the highest concentration in mandarin 26, 10.13 µg/g, and in the lowest concentration in pummelo, 1.71 µg/g (*p* = 0.039). Two sugar acids, glycerol and another identified as 204/333, were found in significantly different concentrations. Glycerol was found in the highest concentration in mandarin 26, 5.06 µg/g, and in the lowest concentration in mandarin 2, 1.97 µg/g (*p* = 0.0267). The sugar acid 204/333 was found in the highest concentration in mandarin 26, 73.84 µg/g, and in the lowest concentration in mandarin 31, 9.22 µg/g (*p* = 0.0247).

Percentage of metabolites categorized by type from the total metabolites found in leaves sampled from each of four Cleopatra Mandarin selections and Pummelo.

### 3.6. Diaphorina citri Oviposition and Survivorship on Mandarin Selections and Pummelo

*Diaphorina citri* showed mixed success in both the number of eggs laid and the survivorship of each egg to adulthood (Table 5). Psyllids were provided with whole plants that had at least ten clusters of young leaf growth, stages #13 to #17 [5] on each stem. Older leaves were present on some stems. This provided a range of ages and ensured that experimental results were not, due to differences in available young leaf tissue. Mandarin 2 and pummelo both had high oviposition levels, but survivorship on mandarin 2 was lower with 236 of the total 578 eggs laid surviving to adulthood (40.83% confidence interval-36.9–44.9%). Psyllids survived to the adult stage at much higher rates on pummelo, with 371 of the 457 eggs laid surviving to the adult stage (81.18%, confidence interval-77.4–85.6%). Oviposition rates and survivorship to adult were both low on mandarin 19 and mandarin 26. Psyllids laid eggs in moderate levels on both selections with 87 eggs found on mandarin 19 and 76 eggs found on mandarin 26. However, in both of these cases, most of the nymphs did not survive past the second instar, with only four psyllids surviving to the adult stage (4.6%, confidence interval-1.5–10.7%) on mandarin 19 and only three surviving to the adult stage (3.95%, confidence interval-1.1–10.3%) on mandarin 26. Interestingly, the highest survival rate was found on mandarin 31, along with the lowest oviposition rate. Psyllids only laid 29 eggs on mandarin 31, and 27 (93%, confidence interval-79.3–85.6%) of these survived to the adult stage.

## 4. Discussion

The EPG results herein outline a range of behavioral responses in *D. citri* probing among mandarin selections and pummelo. While no one selection was consistently the most resistant or the most susceptible to *D. citri* probing, there was a clear trend. The most resistance-associated probing behaviors were exhibited on mandarin 31 and mandarin 2, while a larger number of behaviors that can be associated with susceptibility to *D. citri* probing occurred on mandarin 19 and pummelo, with mandarin 26 being intermediate. The probing profiles of *D. citri* on mandarin selections and pummelo reveal host plant resistance mechanisms encountered at every level of the probing process. Some resistance mechanisms were encountered prior to or at the very onset of probing, at the cuticle or epidermis. Others were encountered within the parenchyma tissues, at the edge of the vascular tissues acting as a barrier to phloem access, while others were encountered within the phloem sap, reducing phloem ingestion durations.

The behavioral profiles of *D. citri* probing some hosts indicate resistance factors encountered early during the probing process, in the cuticle, epidermis or parenchyma tissues. Resistance factors in the cuticle or epidermis are indicated by an increase in time spent not probing or by a time delay to the first probe. Cuticular resistance factors may include volatile compounds in the waxy matrix of the cuticle. *Citrus* and other Rutaceous hosts of *D. citri* have complex, volatile profiles that vary among species, including many forms of terpenes [29,30]. Resistance factors in the epidermis may include oil glands, and silica inclusions called raphides [31].

Resistance factors in parenchyma are indicated by an increased number of initial probes that do not reach phloem or xylem tissues, an increased duration of time spent performing pathway waveforms, or increased time to first ingestion phase. In psyllids, variables associated with increased time spent performing waveform C, with stylets primarily in parenchymal tissues, indicate resistance factors in the parenchyma. Parenchyma tissue resistance factors can include cell walls resistant to the physical and chemical penetration by the pest insect. The parenchyma tissues of wheat, *Triticum aestivum*, cultivars resistant to the aphid, *Schizaphis graminum*, were not easily penetrated during probing. Parenchyma cell walls appeared resistant to aphid pectinase activity [32]. Pectinases are required for insects to successfully probe intercellularly through host plant tissues as pectinases are used to dissolve the middle lamellae between adjacent plant cell walls [33]. Collectively, time spent overcoming these various resistance factors delays the onset of phloem salivation and ingestion and the potential for pathogen acquisition and inoculation.

Additional resistance factors can influence initial access to phloem sieve elements, indicated by long durations of waveforms D and E1—both performed in phloem tissues prior to phloem ingestion. Waveform D has been correlated with stylet tips in phloem, even during very early-interrupted events (6 s into waveform D) [24], and likely represents the initial tasting of the phloem sieve element. Within Rutaceous host species, there can be a barrier of thickened sclerenchymatous phloem fiber cells surrounding the phloem sieve elements. Phloem fiber cells can be absent in young leaves and appear or develop more layers as the leaf ages [5], and the presence or thickness can be variable among species [34]. When present, the phloem fiber cells are a barrier that must be crossed or circumvented in order for *D. citri* to access phloem sieve elements [35,36]. Waveform E1 has been correlated with phloem salivation and functions to overcome chemical and physical defenses within the sieve element prior to initiating phloem ingestion [23,37]. Hemipteran salivary enzymes, such as glucose dehydrogenase, can be used to detoxify plant defensive compounds [38]. Psyllids performed longer durations of phloem salivation (E1) on the two most resistant mandarins, while performing significantly less phloem salivation on the most susceptible plant tested, pummelo. Time spent overcoming the phloem fiber cells delays the onset of phloem salivation and potential pathogen inoculation, while time spent overcoming the initial chemistry of phloem sieve elements delays the onset of and phloem ingestion, and potential pathogen acquisition by the vector.

After phloem sieve elements are contacted and phloem ingestion initiated, resistance factors within the phloem sieve elements influence an insect’s ability to ingest phloem sap or maintain long durations of phloem ingestion on the host plant. These factors are likely tied to the insect’s ability to manage both the secondary metabolite and nutritional chemistry of the phloem sap [39,40,41]. The phloem sap is a nutrient-rich food source, although it can be lacking in essential amino acids, it presents osmotic challenges to probing insects, and could be defended by an array of plant defensive compounds. Essential amino acids lacking in phloem sap can be supplemented by bacterial endosymbionts, and *D. citri* is known to have several important endosymbionts that provide nutritional enhancements [42,43]. The osmotic challenges to phloem sap ingestion can be offset by ingesting from the less nutrient-dense xylem sap [44], and probing trends of *D. citri* suggest they opt to ingest xylem when phloem ingestion cannot be maintained [5,24]. The defensive compounds in the phloem of *Citrus* and other rutaceous hosts include organic acids, fatty acids, and amino acids [45].

In addition to the probing differences observed on the mandarin selections and pummelo, we found differential oviposition and survival trends that roughly mirror the adult probing ability of each host. *Diaphorina citri* oviposited, and eggs and nymphs survived best on the hosts on which the adults probed most readily. Since *D. citri* oviposit and the nymphs develop on the same host range as the adults feed, the oviposition, survival, and probing behavior all contribute to our understanding as to the host suitability for *D. citri*. The oviposition and survival of *D. citri* have been investigated on several *Citrus* and Rutaceous hosts [46,47,48], but no study has thoroughly investigated both the oviposition and survival, and related these data with the probing behavior of adult *D. citri*, as we have done in the current study.

Host plant metabolites play an important role in deterring or minimizing herbivory, and host metabolites have been shown to impact pest insect behavior and host preference in hemipteran pests [49,50,51]. Plant metabolites can be constitutive or induced [52], and can have diverse functions, including: Defense against herbivores or pathogens [53], as a response to mechanical damage [54,55], and as a response to environmental stress [56]. The leaf metabolites found in mandarin selections and pummelo provide additional data on the complex vector-host plant interactions of *D. citri* probing *Citrus*, but do not explain specific aspects of the observed probing differences. This is, in part, due to the incredibly complex nature of how and when plants manage their arsenal of defensive compounds. We do not know which types of metabolites are constitutive and which are induced by *D. citri* probing in each of the plants tested.

We observed interesting trends in the balance of metabolites which are precursor molecules to plant defensive compounds and in the nutritional metabolites found in the mandarin selections and pummelo tested, but no clear explanation as to why *D. citri* probed and ingested from phloem more frequently from some hosts and less from others. The organic acids, succinic and quinic acids, were not necessarily found in the highest levels in selections most resistant to *D. citri* probing. Succunic acid was found in the highest concentration in pummelo, the species most susceptible to *D. citri* probing. The other concentrations of succinic acid do not line up neatly with the resistance spectrum defined by EPG variables and *D. citri* probing behavior. As an organic acid, succinic acid was expected to be found in the highest concentrations in the most resistant mandarin selections, mandarin 31 and 2. In fact, Mandarin 2 was found to contain the highest concentration of quinic acid, with mandarin 19 and 31, containing only slightly lower concentrations, within 0.1 µg/g.

Pummelo contained more than twice concentration of the amino acid serine compared to other selections, with the next highest concentration found in mandarin 26. Pummelo also exhibited three times the concentration of the sugar xylose compared to other selections, with the next highest concentration found in mandarin 26. Pummelo had higher than average concentrations of other sugars and sugar acids compared to the mandarin selections, indicating that pummelo is more nutritious to psyllids compared to the tested mandarin selections. While pummelo is relatively defended against psyllids and other pests, having generally higher levels of succinic acid and quinic acids found in leaf tissues, *D. citri* probed pummelo most successfully compared to the tested mandarin selections, possibly indicating that nutrition is more important to *D. citri* than defense on these tested host plants.

Another interpretation of our results is that pummelo, the most susceptible plant tested, may not have a complex arsenal of constitutive defensive metabolites, but rather upregulated the production of succinic acid and quinic acid in response to *D. citri* probing, while the other more resistant mandarin selections, 31 and 2, had generally high constitutive levels of these compounds. Defensive plant metabolites can be induced in response to pest attack [57], pathogen attack [45], and as a wound response [58]. It is also possible that the behavioral profiles of *D. citri* found on mandarin selections and pummelo is more closely correlated with psyllid nutrition rather than defensive metabolites. Albrecht, et al. [53] found sugar concentrations to be a limiting factor of HLB disease development in Cleopatra mandarin and found defensive compounds were not tightly associated with resistance to HLB. Lower concentrations of sugars and sugar acids in mandarin 31 and mandarin 2 might be more important variables linking *D. citri* probing behavior and host preference rather than high levels of defensive metabolites.

Though not statistically significant in our study, γ-aminobutyric acid (GABA) levels were highest in the mandarin selections most resistant to *D. citri* probing and lowest in the mandarin selection and pummelo most susceptible to *D. citri* probing. There is a growing body of evidence that increased GABA levels act as an antibiosis mechanism of plant defense, negatively impacting insects through reduced feeding and survival [59,60,61]. Insects performed less well on diets enhanced with GABA [62,63]. Transgenic tobacco plants (*Nicotiana tabacum*) that over-produce GABA, deter feeding by tobacco budworms, *Heliothis virescens* [64]. The mechanism of GABA impact on insect neurochemistry is thought to occur through inhibition of GABA-controlled chloride channels in the peripheral nervous system.

## 5. Conclusions

There is much yet to be learned about the complex interactions between plant metabolites, vector probing and transmission behavior, oviposition, and development on *Citrus* hosts. The detailed quantification of *D. citri* probing behavior, oviposition, survivorship, and identification of Cleopatra mandarin hybrid selections more resistant to psyllid probing combined with the detection of many important metabolic compounds summarized in this study provides new insights into *D. citri* host preference, and *Citrus* resistance to vector probing and pathogen transmission, and may contribute to the development of psyllid-resistant rootstock.

## Figures and Tables

**Table 1:** Probing behavior of *Diaphorina citri* on Cleopatra Mandarin selections and Pummelo.

Variable	Unit	Mandarin 2		Mandarin 19		Mandarin 26		Mandarin 31		Pummelo		*p*-Value
DurFrstPrb	hr	0.8 ± 0.295	c	1.9 ± 0.295	a	1 ± 0.295	bc	0.2 ± 0.295	d	1.1 ± 0.295	b	0.0001
DurScndPrb	hr	1.7 ± 0.288	a	1.6 ± 0.288	a	0.8 ± 0.288	c	0.1 ± 0.288	d	1.3 ± 0.288	b	0.0001
TtlPrbTm	hr	17.8 ± 0.406	a	17.6 ± 0.406	a	16.2 ± 0.406	b	14.8 ± 0.406	c	16.6 ± 0.406	b	0.0001
TtlDurNP	hr	4.7 ± 0.411	c	5.1 ± 0.411	c	7 ± 0.411	a	7.9 ± 0.411	a	6.4 ± 0.411	b	0.0001
TmFrstPrbFrmStrt	hr	0.2 ± 0.102	c	0.4 ± 0.099	a	0.3 ± 0.107	ab	0.6 ± 0.104	a	0.2 ± 0.104	c	0.0493
DurNnprbBfrFrstE1	hr	2.9 ± 0.389	b	2.1 ± 0.389	c	3.6 ± 0.389	a	3.8 ± 0.389	a	2.6 ± 0.389	b	0.0001
TtlDurC	hr	11 ± 0.408	a	8.6 ± 0.408	d	10.1 ± 0.408	b	9.4 ± 0.408	c	7.9 ± 0.408	e	0.0001
MnDurC	hr	0.4 ± 0.058	a	0.4 ± 0.057	a	0.4 ± 0.061	a	0.3 ± 0.059	b	0.2 ± 0.059	c	0.0450
PrcntPrbC	%	62 ± 0.210	b	51 ± 0.20	c	62 ± 0.22	b	66 ± 0.21	a	48 ± 0.21	c	0.0502
DurG	hr	3.0 ± 0.326	a	2.0 ± 0.326	b	2.2 ± 0.342	bc	1.4 ± 0.342	d	2.4 ± 0.342	b	0.0159
CtoFrstG	sec	2.6 ± 0.97	e	3.2 ± 0.97	d	4.8 ± 1.02	c	6.8 ± 1.02	b	7.5 ± 1.02	a	0.0016
DurNnprbBfrFrstG	hr	0.7 ± 0.253	c	0.8 ± 0.253	c	1.2 ± 0.265	b	2.2 ± 0.265	a	1 ± 0.265	b	0.0013
NumLngG	#	4.6 ± 0.38	a	3.3 ± 0.38	c	3.0 ± 0.40	d	3.4 ± 0.40	c	3.7 ± 0.40	b	0.0362
TmFrstSusGFrstPrb	hr	1.5 ± 0.327	d	2.7 ± 0.327	bc	2.9 ± 0.327	b	3.5 ± 0.327	a	2.5 ± 0.327	c	0.0001
meanD	sec	66 ± 9.01	c	74 ± 7.62	b	63 ± 8.01	c	90 ± 8.01	a	43 ± 7.80	d	0.0019
DurNnprbBfrFrstD	hr	2.9 ± 0.316	a	2 ± 0.316	b	2.9 ± 0.316	a	2.7 ± 0.316	a	1.8 ± 0.316	b	0.0001
TmFrmFrstPrbFrstD	hr	7.1 ± 0.704	b	6.2 ± 0.704	c	7.8 ± 0.704	a	5.9 ± 0.704	d	6.4 ± 0.704	c	0.0001
NumLngD	#	1.1 ± 0.75	b	2.1 ± 0.64	a	0.3 ± 0.67	c	2.4 ± 0.67	a	0.2 ± 0.65	c	0.0497
TmFrstSusDFrstPrb	hr	14.1 ± 1.099	b	15.4 ± 1.099	a	6.7 ± 1.099	c	13.7 ± 1.099	b	15.5 ± 1.099	a	0.0001
maxD	sec	97.6 ± 22.99	b	129.9 ± 19.43	a	91.5 ± 20.43	b	142 ± 20.43	a	61 ± 19.91	c	0.0426
PrcntPrbD	%	6.2 ± 0.20	c	8.5 ± 0.19	b	6.4 ± 0.21	c	10.3 ± 0.20	a	8.10 ± 0.20	b	0.0503
MnDurE1	sec	76.4 ± 9.68	a	50.2 ± 8.19	b	69 ± 8.61	a	45.6 ± 8.61	c	45.9 ± 8.39	c	0.0463
TmFrmFrstPrbFrstE	hr	12.3 ± 0.775	a	7.5 ± 0.775	c	8.6 ± 0.775	b	7.5 ± 0.775	c	7.2 ± 0.775	c	0.0001
DurFirstE	hr	1.1 ± 0.448	b	1.9 ± 0.379	a	0.7 ± 0.398	c	0.7 ± 0.398	c	1.9 ± 0.388	a	0.0501
NumLngE2	#	1.3 ± 0.46	d	2.7 ± 0.45	b	2.2 ± 0.49	c	2.4 ± 0.47	bc	3.6 ± 0.47	a	0.0175
TtlDurE2	hr	6 ± 0.392	b	10.4 ± 0.392	a	4.8 ± 0.392	c	4.7 ± 0.392	c	6.8 ± 0.392	b	0.0001
TmFrstSusE2FrstPrb	hr	15 ± 0.8	a	8.8 ± 0.8	c	11.2 ± 0.8	b	11.2 ± 0.8	b	7.4 ± 0.8	d	0.0001
TmFrstE2StrtEPG	hr	13.8 ± 0.8	a	9.1 ± 0.8	c	10.9 ± 0.8	b	10.8 ± 0.8	b	7.4 ± 0.8	d	0.0001
TmFrstE2FrmFrstPrb	hr	13.6 ± 0.793	a	8.7 ± 0.793	c	10.7 ± 0.793	b	10.2 ± 0.793	b	7.2 ± 0.793	d	0.0001
TmLstE2EndRcrd	hr	5.6 ± 0.965	b	2.6 ± 0.965	c	5.9 ± 0.965	b	8.3 ± 0.965	a	1.9 ± 0.965	c	0.0001
maxE2	hr	3.5 ± 0.547	b	4.8 ± 0.509	a	2.8 ± 0.493	c	2.3 ± 0.478	c	3 ± 0.452	bc	0.0081
PrcntPrbE2	%	29 ± 0.41	b	57 ± 0.38	a	19.5 ± 0.37	c	22.5 ± 0.36	c	37 ± 0.34	b	0.0149
PrcntE2SusE2	%	44 ± 0.35	b	69 ± 0.29	a	72 ± 0.33	a	50 ± 0.27	b	72 ± 0.27	a	0.0184
TtlDurE	hr	5.3 ± 0.441	c	7.6 ± 0.441	a	4.2 ± 0.441	d	4.3 ± 0.441	d	6.6 ± 0.441	b	0.0001
TtlDurE1FllwdE2PlsE2	hr	6.1 ± 0.395	b	10.5 ± 0.395	a	4.9 ± 0.395	c	4.7 ± 0.395	c	6.9 ± 0.395	b	0.0001
TtlDurNnPhlPhs	hr	17.3 ± 0.432	b	15.3 ± 0.432	d	19.1 ± 0.432	a	18.4 ± 0.432	a	16.4 ± 0.432	c	0.0001
TmFrstSusE2	hr	15.1 ± 0.79	a	9.1 ± 0.79	c	11.4 ± 0.79	b	11.6 ± 0.79	b	7.5 ± 0.79	d	0.0001

Data are means and standard errors generated by ANOVA using a SAS program from Ebert et al., 2015. Eighty-nine variables were calculated and thirty-seven were found to be statistically different and are summarized. Means with different letter designations are statistically different from one another. *p* = 0.05.

**Table 2:** Continuous ranks of probing variables of *Diaphorina citri* probing four Mandarin selections and Pummelo.

	Variable	Mandarin 2	Mandarin 19	Mandarin 26	Mandarin 31	Pummelo
1	TtlDurNP	1	2	4	5	3
2	TmFrstPrbFrmStrt	1	4	3	5	1
3	DurNnprbBfrFrstE1	3	1	4	5	2
4	TtlDurC	5	2	4	3	1
5	PrcntPrbC	4	2	3	5	1
6	DurG	5	2	3	1	4
7	NumLngD	3	4	2	5	1
8	maxD	3	4	2	5	1
9	meanD	3	4	2	5	1
10	DurNnprbBfrFrstD	5	2	4	3	1
11	TmFrmFrstPrbFrstD	4	2	5	1	3
12	PrcntPrbD	1	4	2	5	3
13	TtlDurNnPhlPhs	3	1	5	4	2
14	TmFrstSusE2	5	2	3	4	1
15	TmFrmFrstPrbFrstE	5	2	4	3	1
16	TmFrstSusE2FrstPrb	5	2	4	3	1
17	TmFrstE2StrtEPG	5	2	4	3	1
18	TmFrstE2FrmFrstPrb	5	2	4	3	1
19	TmLstE2EndRcrd	3	2	4	5	1
20	maxE2	2	1	4	5	3
21	PrcntPrbE2	3	1	5	4	2
22	PrcntE2SusE2	5	3	1	4	2
23	NumLngE2	5	2	4	3	1
24	TtlDurE2	3	1	4	5	2
	Column Sum	87	54	84	94	40
	Rank	4	2	3	5	1
					Most Resis.	Most Susc.

Means of twenty-four electropenetrography (EPG) variables statistically significant between treatments (*p* = 0.05) were ranked along a continuum from one to five, the ranks summed, and a resistance rank assigned to each host plant tested.

**Table 3:** Discriminant analysis matrix showing differences in *Diaphorina citri* probing responses to four Cleopatra Mandarin selections and Pummelo.

	Mandarin 2	Mandarin 19	Mandarin 26	Mandarin 31	Pummelo
Mandarin 2	0	2.43925	1.0376	4.44439	3.27002
Mandarin 19	0.0037	0	2.35387	5.59795	5.97433
Mandarin 26	0.2727	0.0069	0	1.85684	1.89187
Mandarin 31	0.0001	0.0001	0.038	0	5.55242
Pummelo	0.0004	0.0001	0.0345	0.0001	0

The upper triangular matrix (with a light gray background) is the Mehalanobis distances between *D. citri* behavior on two compared hosts. Larger values in the upper triangle represent larger differences between the compared treatments calculated from the eight variables used to create the matrix. The lower triangular matrix is a *p*-value testing the null hypothesis that the distance between the compared behaviors is zero. *p* = 0.05.

**Table 4:** Metabolites found in leaf tissue of Cleopatra Mandarin selections and Pummelo.

	Metabolite	Mandarin 2	Mandarin 19	Mandarin 26	Mandarin 31	Pummelo	*p*-Value
1	Ferulic Acid 338	7.06 ± 6.81	26.87 ± 32.38	45.78 ± 40.40	33.77 ± 37.14	7.76 ± 8.95	0.096
2	Gluconic Acid	11.26 ± 14.76	7.33 ± 7.70	31.29 ± 30.03	4.06 ± 3.21	3.90 ± 6.62	0.7348
3	Citric Acid	3989 ± 5276	2316 ± 4308	5901 ± 4018	3287 ± 6973	8546 ± 11,588	0.23
4	Quinic Acid	9.75 ± 0.84 a	9.09 ± 0.84 a	4.17 ± 0.84 b	9.08 ± 0.84 a	8.48 ± 0.84 a	**0.0203**
5	Malic Acid	729.46 ± 1459.26	119.33 ± 165.17	275.41 ± 280.76	1,465.81 ± 869.77	422.74 ± 249.38	0.0864
6	Synephrine	228.52 ± 365.65	274.02 ± 357.05	246.44 ± 337.61	141.95 ± 218.21	0	0.3256
7	Succinic Acid	4.24 ± 2.46 ab	1.18 ± 2.46 b	4.49 ± 2.46 ab	3.43 ± 2.46 ab	12.99 ± 2.46 a	**0.0297**
8	Fumaric Acid	0.84 ± 1.10	2.41 ± 4.02	2.98 ± 3.39	1.22 ± 0.94	1.22 ± 1.24	0.834
9	Phosphoric Acid	13.76 ± 19.06	0	0	0	66.54 ± 17.25	0.2055
10	Maleic Acid	21.86 ± 22.15	20.79 ± 24.03	110.88 ± 144.76	76.40 ± 59.14	129.57 ± 149.87	0.3013
11	Lactic Acid	4.00 ± 7.89	8.90 ± 13.51	26.46 ± 53.07	10.24 ± 15.38	5.85 ± 5.52	0.7579
12	Oxalic Acid	4.14 ± 2.66	2.46 ± 3.04	3.92 ± 3.3	52.28 ± 88.07	27.03 ± 50.68	0.3798
13	Palmitic Acid	171.63 ± 146.79	62.77 ± 131.28	204.87 ± 250.98	172.55 ± 209.70	45.40 ± 33.12	0.4819
14	Oleic Acid	1.11 ± 0.72 bc	2.44 ± 0.72 a	1.85 ± 0.72 ab	0.4 ± 0.72 abc	1.47 ± 0.8 c	**0.0072**
15	Stearic Acid	4.27 ± 2.47	2.82 ± 4.78	18.59 ± 21.38	120.75 ± 256.19	19.72 ± 26.09	0.327
16	L-Alanine	24.96 ± 25.13	22.81 ± 20.35	2.84 ± 2.01	22.08 ± 36.15	43.70 ± 77.58	0.3133
17	Glutamic Acid	8.13 ± 11.47	0.97 ± 1.11	23.93 ± 41.10	22.07 ± 36.68	77.57 ± 95.77	0.6388
18	L-Aspartic Acid	18.05 ± 31.34	5.03 ± 7.42	19.66 ± 17.27	8.77 ± 9.81	20.48 ± 22.38	0.8808
19	γ- Aminobutyric Acid	360.04 ± 664.31	590.50 ± 715.28	725.34 ± 717.59	974.47 ± 529.83	79.91 ± 93.21	0.6335
20	L-Threonine	12.69 ± 9.53	10.16 ± 13.50	41.98 ± 57.13	18.63 ± 21.13	35.38 ± 35.41	0.6296
21	Serine	24.21 ± 36.44 b	18.02 ± 36.44 b	76.78 ± 36.44 ab	26.26 ± 36.44 b	187.76 ± 36.44 a	**0.0173**
22	L-Isoleucine	5.28 ± 3.35	2.45 ± 3.85	2.15 ± 4.21	1.29 ± 0.54	6.75 ± 6.69	0.3506
23	L-Proline	155.95 ± 111.91	13.57 ± 9.52	40.55 ± 26.16	10.80 ± 8.45	128.26 ± 206.64	0.0669
24	Glycine	0	1.93 ± 2.38	20.81 ± 17.85	25.00 ± 35.82	18.09 ± 17.96	0.2206
25	L-Valine	2.62 ± 2.28	2.82 ± 4.78	18.59 ± 21.38	4.02 ± 7.02	1.10 ± 1.06	0.324
26	2-Aminopropanol	37.54 ± 38.67	52.81 ± 36.33	51.34 ± 41.49	15.73 ± 4.03	11.93 ± 11.12	0.297
27	Xylose 1	3.37 ± 2.35 b	4.15 ± 2.35 b	6.30 ± 2.35 b	3.08 ± 2.35 b	16.78 ± 2.35 a	**0.0473**
28	Xylose 2	48.96 ± 58.79	13.06 ± 17.08	32.76 ± 58.84	36.09 ± 48.58	45.15 ± 43.79	0.8393
29	Arabinose	1.50 ± 1.04	7.56 ± 5.72	4.46 ± 3.34	5.25 ± 6.92	3.77 ± 2.01	0.5822
30	Erythrose	156.54 ± 78.21	239.49 ± 64.14	254.54 ± 100.14	260.12 ± 119.96	181.15 ± 5.04	0.2415
31	Fructose	414.94 ± 471.91	648.13 ± 864.54	2827 ± 4297	1183 ± 1404	1162 ± 1412	0.3747
32	Mannose	104.01 ± 177.97	128.47 ± 149.63	140.10 ± 125.65	192.22 ± 103.78	59.57 ± 71.99	0.433
33	Glucose	7001 ± 12025	1569 ± 2011	5770 ± 12,120	1037 ± 1240	2398 ± 4143	0.9915
34	Glucopyranose	11.19 ± 16.33	32.25 ± 35.77	19.94 ± 21.38	18.18 ± 15.79	3.29 ± 3.95	0.3198
35	Threose	8.13 ± 11.20	12.64 ± 15.14	17.22 ± 10.86	19.24 ± 13.40	8.61 ± 10.88	0.6122
36	Pyranoside 204/338	1.18 ± 0.44	5.16 ± 6.12	1.15 ± 1.39	0.16 ± 0.06	0.24 ± 0.45	0.8232
37	α-galactoside	5.06 ± 1.93 ab	2.95 ± 1.93 ab	10.13 ± 1.93 a	2.50 ± 1.93 ab	1.71 ± 1.93 b	**0.039**
38	Deoxy-galactoside	393.40 ± 869.20	74.87 ± 102.35	59.01 ± 43.88	19.72 ± 25.23	8.58 ± 15.46	0.2983
39	Glucoheptose	932 ± 1318	1350 ± 1316	1300 ± 2053	1404 ± 2826	356.07 ± 566.44	0.2401
40	Sucrose	24,586 ± 20,814	10,144 ± 9511	26,319 ± 56,240	11,322 ± 15,132	10,305 ± 10,812	0.1514
41	Maltose	66.46 ± 50.97	332.82 ± 376.90	31.92 ± 26.38	69.59 ± 54.02	229.71 ± 157.41	0.7644
42	Unk Disaccharide 361	218.88 ± 222.90	94.45 ± 152.11	188.48 ± 340.13	283.68 ± 261.95	67.99 ± 111.31	0.1241
43	Unk Disaccharide	87.59 ± 69.97	11.82 ± 12.26	19.89 ± 20.037	53.94 ± 50.23	69.23 ± 86.47	0.5592
44	Inositol-2- Phosphate	15.89 ± 21.34	10.65 ± 7.55	18.13 ± 23.89	11.55 ± 14.80	1.79 ± 1.86	0.946
45	Scyllo- Inositol	218.96 ± 167.70	193.23 ± 156.54	326.86 ± 411.38	281.57 ± 189.51	197.42 ± 256.13	0.7775
46	Myo- Inositol	351.59 ± 546.51	2.46 ± 3.04	3.92 ± 3.30	93.55 ± 184.35	205.76 ± 227.12	0.1965
47	Phytol 143	13.94 ± 18.45	0	45.89 ± 56.93	9.72 ± 3.68	0.02 ± 0.04	0.3732
48	Glycerol	1.97 ± 0.67 b	4.9 ± 0.67 a	5.06 ± 0.67 a	4.32 ± 0.67 ab	4.46 ± 0.67 ab	**0.0267**
49	Xylitol	87.91 ± 181.95	252.24 ± 251.94	382.21 ± 346.67	110.18 ± 137.42	117.65 ± 150.62	0.318
50	Glucitol	11.87 ± 16.82	5.53 ± 6.40	7.27 ± 10.61	3.35 ± 3.11	0.96 ± 1.57	0.6759
51	Mannitol	34.73 ± 38.52	37.67 ± 55.35	48.29 ± 39.73	17.44 ± 21.41	97.69 ± 108.58	0.3695
52	Chiro-Inositol	730.71 ± 818.83	1337 ± 1033	1334 ± 2109	803.14 ± 720.79	0	0.4593
53	Sugar Alcohol 217/319	6.17 ±4.01	5.78 ± 6.52	11.96 ± 10.08	11.03 ± 8.31	3.31 ± 5.84	0.4336
54	Ribonic Acid	93.48 ± 112.08	34.67 ± 43.88	64.91 ± 78.58	140.54 ± 121.14	71.06 ± 78.88	0.2806
55	Saccharic Acid	14.12 ± 24.50	5.88 ± 4.38	20.83 ± 23.03	12.21 ± 21.97	8.61 ± 5.06	0.6895
56	Sugar Acid 204/333	17.10 ± 14.16 b	19.12 ± 14.16 b	73.84 ± 14.16 a	9.22 ± 14.16 b	14.37 ± 14.16 b	**0.0247**
57	Glucuronic Acid	3.37± 3.71	2.59 ± 2.88	7.06 ± 11.11	6.43 ± 5.96	1.19 ± 2.67	0.5053
58	Threonic Acid Deriv.	66.70 ± 123.42	15.65 ± 15.43	33.12 ± 22.20	67.12 ± 81.69	31.79 ± 37.23	0.1031
59	Threonic Acid	48.34 ± 34.19	193.47 ± 193.11	95.82 ± 65.08	100.25 ± 114.15	974.05 ± 1182.63	0.8799
60	2-Ketoglutaric Acid	2.04 ± 2.02	2.24 ± 0.75	2.08 ± 2.61	6.50 ± 7.55	5.70 ± 5.44	0.5845
61	Arabino- Hexaric Acid	5.59 ± 5.12	7.28 ± 10.57	5.44 ± 2.67	3.30 ± 3.88	4.52 ± 3.49	0.5592
62	Unk Sugar Acid 333	75.24 ± 92.05	26.66 ± 38.38	132.95 ± 202.05	165.58 ± 185.82	34.11 ± 42.22	0.2685
63	Glycerol Glycoside	170.89 ± 251.21	256.32 ± 375.03	180.57 ± 177.95	33.23 ± 55.60	34.83 ± 27.99	0.3235

Metabolites are presented as µg/g. Data are means and standard errors generated by ANOVA using SAS. Means with different letter designations are statistically different from one another and displayed in bold text. *p* = 0.05.

**Table 5:** Oviposition and survival of *Diaphorina citri* on four Mandarin selections and Pummelo.

Selection/Species	Eggs Laid	Adults Counted				Total Adults Emerged	% Adults Emerged from Egg (95% CI)
	Day 10 ^1^	Day 20 ^2^	Day 24	Day 27	Day 29		
Mandarin 2	578	12	139	66	19	236	40.83% (36.9–44.9%) b
Mandarin 19	87	0	3	1	0	4	4.60% (1.5–10.7%) c
Mandarin 26	76	0	0	3	0	3	3.95% (1.1–10.3%) c
Mandarin 31	29	2	3	6	6	27	93.10% (79.3–100%) a
Pummelo	457	42	241	78	11	371	81.18% (77.4–85.6%) a

^1^ Adults were removed at day 10 and as they emerged to avoid additional oviposition. ^2^ First adults emerge. CI = Confidence interval. Letters designate statistically different percent survival to adult form, *p* = 0.05.

## Abbreviations

CtoFrstG	The number of probes to first waveform xylem sap ingestion event
DurFirstE	Duration of first E event (mean)- may include phloem salivation (E1) only or include phloem ingestion (E2) if the first E2 event immediately follows the first E1.
DurFrstPrb	Duration of the first probe (mean)
DurG	Mean duration of the xylem ingestion waveform (G)
DurNnprbBfrFrstD	Cumulative duration of all non-probing events before first phloem contact waveform (D) event
DurNnprbBfrFrstE1	Cumulative duration of all non-probing events before first phloem salivation event (E1)
DurNnprbBfrFrstG	Cumulative duration of all non-probing events before first xylem ingestion event (G)
DurScndPrb	Duration of the second probe
maxD	Longest waveform D event
maxE2	Longest waveform E2 event
meanD	Average duration of waveform D events
MnDurC	Mean duration of pathway waveform (C)
MnDurE1	Mean duration of E1 waveform events
NumLngD	Number of long (100+ seconds) phloem contact (D) events
NumLngE2	Number of long (10+ minutes) E2 waveform events
NumLngG	Number of long (10+ minutes) G waveform events
PrcntPrbC	Of the cumulative time spent in a probe, the percentage of time spent in pathway (waveform C)
PrcntPrbD	Of the cumulative time spent in a probe, the percentage of time spent in phloem contact (waveform D)
PrcntPrbE2	Of the cumulative time spent in a probe, the percentage of time spent in phloem ingestion (waveform E2)
PrcntE2SusE2	Of all time spent ingesting phloem, the proportion of that time spent as sustained (>10 minutes) events.
TmFrmFrstPrbFrst D	Time from first probe to first D waveform event (mean)
TmFrmFrstPrbFrstE	Time from first probe to first E waveform event (mean)
TmFrstE2FrmFrstPrb	Time from first probe to first E2 waveform event (mean)
TmFrstE2StrtEPG	Time to first E2 waveform event from start of recording (mean)
TmFrstPrbFrmStrt	Time to first probe from start of recording (mean) Also equals the duration of first non-probing event.
TmFrstSusDFrstPrb	Time to first sustained D from first probe (mean)
TmFrstSusE2	Time to first sustained waveform E2 event (mean)
TmFrstSusE2FrstPrb	Time from first probe to first sustained (>10 minutes) E2 waveform event
TmFrstSusGFrstPrb	Time from first probe to first sustained (>10 minutes) xylem ingestion (G) waveform event
TmLstE2EndRcrd	Time from the last E2 to end of recording.
TtlDurC	Total duration of C waveform events
TtlDurE	Total duration spent performing both E1 and E2 waveforms
TtlDurE1FllwdE2PlsE2	Total duration of waveform E1 when followed by E2 plus the duration of all E2 events
TtlDurE2	Total duration of E2 waveform events
TtlDurNnPhlPhs	Total duration of non-phloem phase, recording time less the time spent in E1 or E2.
TtlDurNP	Total duration of time spent not probing (mean)
TtlPrbTm	Total probing time
